# Mff functions with Pex11pβ and DLP1 in peroxisomal fission

**DOI:** 10.1242/bio.20135298

**Published:** 2013-08-14

**Authors:** Akinori Itoyama, Satoru Michiyuki, Masanori Honsho, Taizo Yamamoto, Ann Moser, Yumi Yoshida, Yukio Fujiki

**Affiliations:** 1 Graduate School of Systems Life Sciences, Faculty of Sciences, Kyushu University Graduate School, 6-10-1 Hakozaki, Higashi-ku, Fukuoka 812-8581, Japan; 2 Department of Biology, Faculty of Sciences, Kyushu University Graduate School, 6-10-1 Hakozaki, Higashi-ku, Fukuoka 812-8581, Japan; 3 The Hugo W. Moser Research Institute, Kennedy Krieger Institute, John Hopkins University, Baltimore, MD 21205, USA

**Keywords:** Peroxisome morphogenesis, Elongation, Fission, Division, Mitochondrial fission factor, Dynamin-like protein 1, Peroxin Pex11p, Fis1

## Abstract

Peroxisomal division comprises three steps: elongation, constriction, and fission. Translocation of dynamin-like protein 1 (DLP1), a member of the large GTPase family, from the cytosol to peroxisomes is a prerequisite for membrane fission; however, the molecular machinery for peroxisomal targeting of DLP1 remains unclear. This study investigated whether mitochondrial fission factor (Mff), which targets DLP1 to mitochondria, may also recruit DLP1 to peroxisomes. Results show that endogenous Mff is localized to peroxisomes, especially at the membrane-constricted regions of elongated peroxisomes, in addition to mitochondria. Knockdown of *MFF* abrogates the fission stage of peroxisomal division and is associated with failure to recruit DLP1 to peroxisomes, while ectopic expression of *MFF* increases the peroxisomal targeting of DLP1. Co-expression of *MFF* and *PEX11β*, the latter being a key player in peroxisomal elongation, increases peroxisome abundance. Overexpression of *MFF* also increases the interaction between DLP1 and Pex11pβ, which knockdown of *MFF*, but not Fis1, abolishes. Moreover, results show that Pex11pβ interacts with Mff in a DLP1-dependent manner. In conclusion, Mff contributes to the peroxisomal targeting of DLP1 and plays a key role in the fission of the peroxisomal membrane by acting in concert with Pex11pβ and DLP1.

## Introduction

Peroxisome is a ubiquitous, spherical organelle present in virtually all eukaryotes, from yeast to mammals. The growth and division model of peroxisome biogenesis predicts that peroxisomes grow and multiply by taking up newly synthesized proteins from the cytosol (Lazarow and Fujiki, 1985).

Peroxisomal division comprises three stages: elongation, constriction, and fission (Itoyama et al., 2012; Koch et al., 2003; Li and Gould, 2003; Schrader et al., 1998). Pex11p is a peroxisome-specific division factor conserved from yeast to humans (Thoms and Erdmann, 2005). High-level expression of *PEX11* promotes the proliferation of peroxisomes (Marshall et al., 1995; Schrader et al., 1998), while deletion of *PEX11* reduces the number of peroxisomes (Erdmann and Blobel, 1995; Li et al., 2002b), thereby suggesting that Pex11p plays a key role in peroxisomal division. Pex11p also functions in peroxisomal elongation, which is the first step in peroxisomal division (Marshall et al., 1995; Opaliński et al., 2011; Schrader et al., 1998). In mammalian cells, three isoforms have been identified: *PEX11α* (Abe et al., 1998; Li et al., 2002a), *PEX11β* (Abe and Fujiki, 1998; Li et al., 2002b; Schrader et al., 1998), and *PEX11γ* (Li et al., 2002a; Tanaka et al., 2003). *PEX11β* is expressed in almost all types of human cells (Schrader et al., 1998), in contrast to *PEX11α* and *PEX11γ*, which are expressed in a tissue-specific manner (Li et al., 2002a; Schrader et al., 1998), thus strongly suggesting that Pex11pβ plays a fundamental role in peroxisome division.

Dynamin-like protein 1 (DLP1), a member of the large GTPase family, promotes the maintenance of peroxisomal and mitochondrial morphology, especially during membrane fission (Ishihara et al., 2009; Tanaka et al., 2006; Waterham et al., 2007). DLP1 is predicted to mediate the fission of peroxisomes and mitochondria via the formation of large multimeric spirals, in a molecular machinery similar to that of dynamin at the site of endocytosis (Danino and Hinshaw, 2001; Ford et al., 2011; Zhang and Hinshaw, 2001). DLP1 and dynamin have several common multidomains including the GTPase, middle, and GTPase effector domains. In particular, the middle domain functions in the higher-order assembly of both proteins, which is required for the formation of functional multimeric spirals (Ingerman et al., 2005; Ramachandran et al., 2007). Therefore, mutations in the DLP1 middle domain result in the abnormal elongation of peroxisomes and hypertubulation of mitochondria (Tanaka et al., 2006; Waterham et al., 2007). Translocation of DLP1 from the cytosol to peroxisomes and mitochondria is a prerequisite for membrane fission.

Fission1 (Fis1) and mitochondrial fission factor (Mff) are thought to be involved in the peroxisomal targeting of DLP1 in mammalian cells (Gandre-Babbe and van der Bliek, 2008; Kobayashi et al., 2007; Koch et al., 2005; Otera et al., 2010). Fis1 is a tail-anchored protein that functions in the fission of peroxisomes and mitochondria (Kobayashi et al., 2007; Koch et al., 2005). Fis1 interacts with DLP1 and ectopic expression of Fis1 increases the interplay between Pex11pβ and DLP1 (Kobayashi et al., 2007), suggesting that Fis1 recruits DLP1 to peroxisomes. Furthermore, Pex11pβ, Fis1, and DLP1 coordinately regulate the fission step of peroxisomal division (Kobayashi et al., 2007). Meanwhile, Mff, another tail-anchored protein, is involved in the maintenance of peroxisomal and mitochondrial morphology (Gandre-Babbe and van der Bliek, 2008). A recent study reported that the mitochondrial targeting of DLP1 was mediated via direct binding of Mff (Otera et al., 2010), and Mff was recently found to be involved in Pex11p-mediated peroxisomal fission (Koch and Brocard, 2012); however, the precise function of Mff in peroxisomal division remains unclear.

The present study shows that Mff recruits DLP1 to peroxisomes and suggests that a functional complex comprising Pex11pβ, Mff, and DLP1 promotes Mff-mediated fission during peroxisomal division.

## Results

### Dual localization of Mff to peroxisomes and mitochondria

**Fig. 1.**
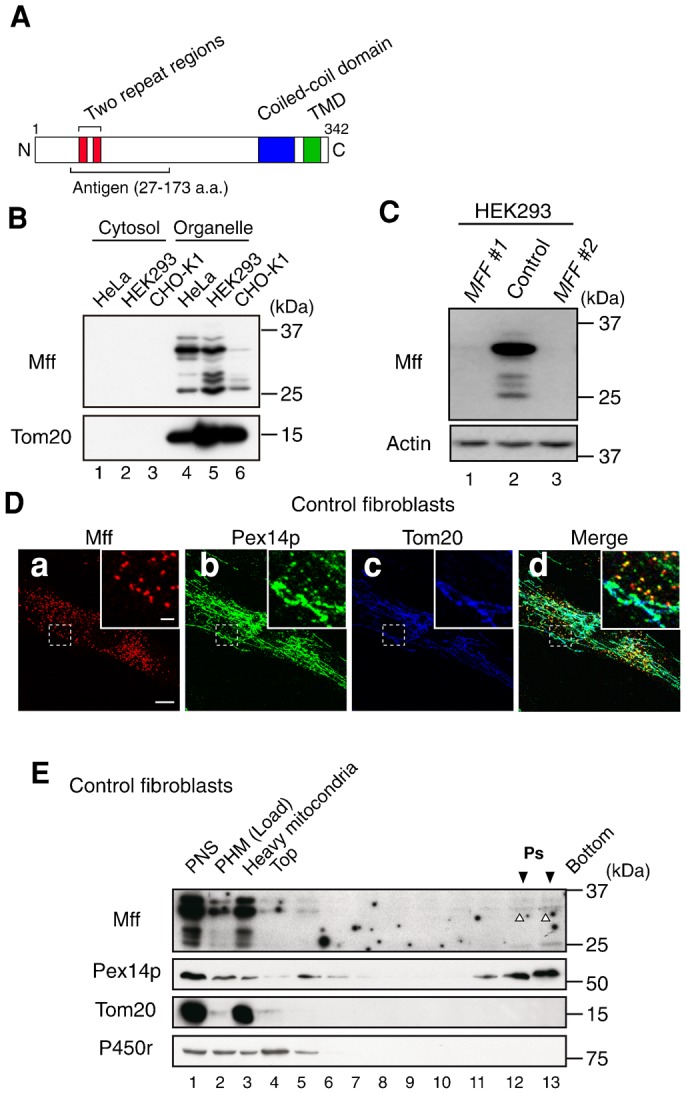
Mff is localized to peroxisomes and mitochondria. (**A**) The domain structure of human Mff splicing variant 8 is presented. The red, blue, and green boxes indicate the two repeat regions, coiled-coil domain and TMD, respectively. The N-terminal 27–173 amino acid portion of human Mff splicing variant 8 was used as an antigen to raise rabbit anti-Mff polyclonal antibody. (**B**) Cytosol and organelle fractions prepared from HeLa, HEK293, and CHO-K1 cells were analyzed by SDS-PAGE and immunoblotting using antibodies to Mff and Tom20. (**C**) HEK293 cells were treated for 72 h with two different dsRNAs (*MFF* #1 and *MFF* #2). Mff levels were assessed by immunoblotting with anti-Mff antibody. Actin was used as a loading control. (**D**) Control fibroblasts were stained with antibodies to Mff (**a**), Pex14p (**b**), and Tom20 (**c**); the merged view of the three proteins is shown (**d**). Scale bar: 10 µm. Insets, higher magnification images of the boxed regions, scale bar: 2 µm. (**E**) PHM fraction from control fibroblasts was fractionated by Opti-prep density gradient ultracentrifugation. The distribution of peroxisomes, mitochondria, and smooth ER was assessed by immunoblotting using antibodies to the marker proteins Pex14p, Tom20, and P450 reductase (P450r), respectively. Downward solid arrowheads indicate the peak fractions of peroxisomes; the upward open arrowhead indicates Mff (Ps; lane 12 and 13).

To investigate the function of Mff, rabbit polyclonal antiserum was raised against the N-terminal region of human Mff splicing variant 8 (residues 27–173) (Fig. 1A). Western blot analysis revealed that the Mff antibody specifically recognized the endogenous Mff protein in organelle fractions from HeLa, HEK293, and Chinese hamster ovary (CHO) cells (Fig. 1B); several bands were detected, including six bands in HEK293 cells. All bands were eliminated by the transfection of siRNA targeting *MFF* (Fig. 1C), possibly reflecting some of the nine Mff splicing variants previously reported (Gandre-Babbe and van der Bliek, 2008).

The subcellular localization of endogenous Mff was investigated by immunostaining with Mff-specific antibody. In control fibroblasts, Mff was mostly localized to Tom20-positive mitochondria and Pex14p-positive peroxisomes (Fig. 1D). In addition, the localization of endogenous Mff was also assessed in post-heavy mitochondrial fractions obtained from control fibroblasts by isopycnic ultracentrifugation (Fig. 1E). Mff was detected in Pex14p-positive peroxisomal fractions (lanes 12 and 13, open arrowheads), which were devoid of Tom20-positive mitochondria or P450r-positive smooth microsomes. Collectively, these results strongly suggest that Mff is localized to peroxisomes as well as mitochondria.

### Mff is essential for peroxisome membrane fission

**Fig. 2.**
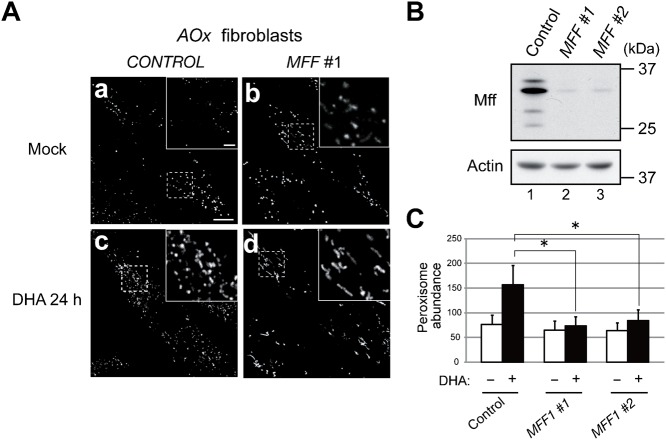
Knockdown of *MFF* abrogates DHA-mediated peroxisome division. (**A**) *AOx*-deficient fibroblasts were treated for 48 h with control dsRNA (left panel) or *MFF* #1 dsRNA (right panel). Cells were further cultured for 24 h in the absence (**a**,**b**) or presence (**c**,**d**) of 150 µM DHA and then stained with anti-Pex14p antibody. Scale bar: 10 µm. Insets, higher magnification images of the boxed regions, scale bar: 2 µm. (**B**) *AOx*-deficient fibroblasts were treated for 48 h with control dsRNA or two different dsRNAs (*MFF* #1 and *MFF* #2). Mff levels were assessed by immunoblotting with anti-Mff antibody. Actin was used as a loading control. (**C**) Peroxisome abundance per cell was measured. Data represent the means ± S.D. of three independent experiments. *P<0.01.

Mff was suggested to be involved in the division of peroxisomes (Gandre-Babbe and van der Bliek, 2008; Otera et al., 2010). To clarify the functional role of Mff in peroxisomal division, the effect of *MFF* knockdown on the division of peroxisomes was assessed in fibroblasts deficient in *acyl-CoA oxidase 1* (*AOx*) encoding the enzyme catalyzing the first step in peroxisomal β-oxidation. We recently reported that docosahexaenoic acid (DHA, C22:6n-3) induces the division of peroxisomes in cells defective in peroxisomal β-oxidation in a Pex11pβ-dependent manner (Itoyama et al., 2012). This is a useful physiological system for inducing peroxisome proliferation. Seventy-two hours after adding *MFF* dsRNA, the Mff protein level was significantly reduced in *AOx*-deficient fibroblasts (Fig. 2B). Peroxisome abundance was greater in *AOx*-deficient fibroblasts treated with control dsRNA and supplemented with DHA (157±39) than in mock-treated cells (76±19). By contrast, DHA-inducible peroxisomal division was strongly inhibited by *MFF* knockdown in two independent experiments using dsRNA *MFF#1* (74±18) and *MFF#2* (84±22), respectively, rather giving rise to numerous elongated peroxisomes (Fig. 2Ac,d,C). These results strongly demonstrate that Mff is essential to peroxisome membrane fission.

### Mff is involved in the recruitment of DLP1 to peroxisomes

**Fig. 3.**
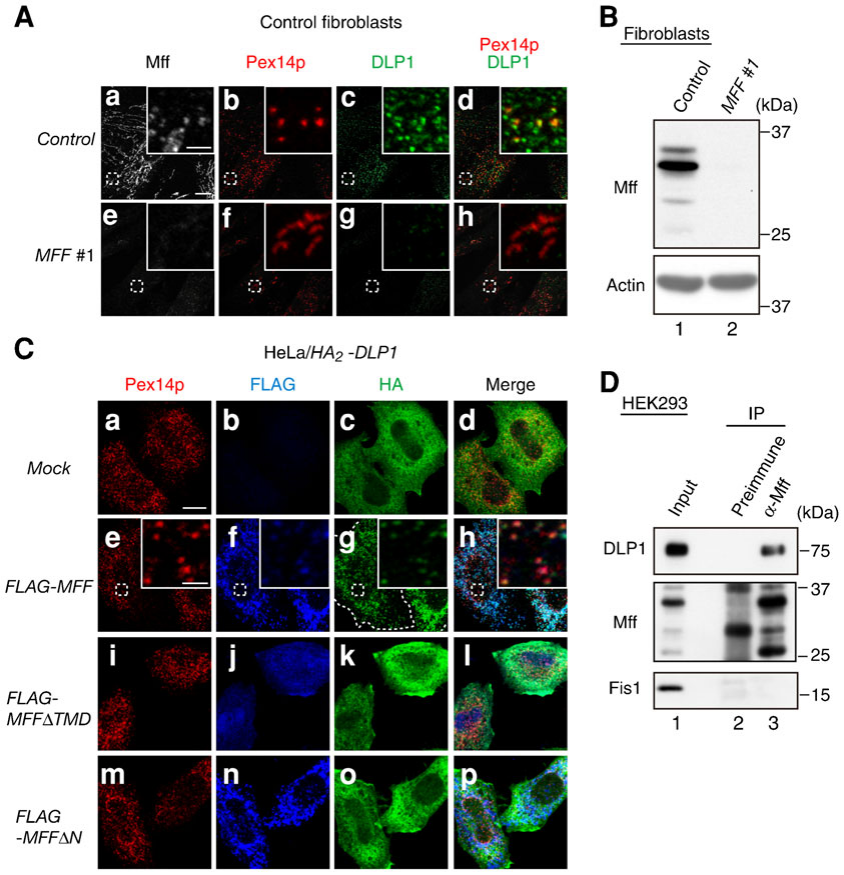
Mff recruits DLP1 to peroxisomes. (**A**) Control fibroblasts were treated for 72 h with control dsRNA (upper panel) or *MFF* #1 dsRNA (lower panel) and then stained with antibodies to Mff (**a**,**e**), Pex14p (**b**,**f**), and DLP1 (**c**,**g**); the merged view of Pex14p and DLP1 is shown (**d**,**h**). Scale bar: 10 µm. Insets, higher magnification images of the boxed regions, scale bar: 2 µm. (**B**) Control fibroblasts were treated for 72 h with control dsRNA or *MFF* #1 dsRNA. Mff levels were assessed by immunoblotting with anti-Mff antibody. Actin was used as a loading control. (**C**) In HeLa cells, *HA_2_*-*DLP1* was expressed (**a–d**) or co-expressed with *FLAG-MFF* (**e–h**), *FLAG-MFFΔTMD* (**i–l**), or *FLAG-MFFΔN* (**m–p**). After 24 h, cells were stained with antibodies to Pex14p (a,e,i,m), FLAG (b,f,j,n) and HA (c,g,k,o); the merged view of the three proteins is shown (d,h,l,p). Scale bar: 10 µm. Insets, higher magnification images of the boxed regions, scale bar: 2 µm. (**D**) HEK293 cells were treated with 0.5 mM DSP and subjected to immunoprecipitation using Mff antiserum (α-Mff, lane 3) or a preimmune serum (preimmune, lane 2). Immunoprecipitates were analyzed by SDS-PAGE and immunoblotting with antibodies to DLP1, Mff, and Fis1. Input (5%) was loaded in lane 1.

Mff functions in the mitochondrial recruitment of DLP1 (Otera et al., 2010). To investigate the potential involvement of Mff in the peroxisomal recruitment of DLP1, the intracellular localization of DLP1 was assessed upon *MFF* knockdown in fibroblasts from a healthy control. Knocking down *MFF* in control fibroblasts significantly reduced the Mff level (Fig. 3B). In cells treated with control RNAi, DLP1 was observed as dot-like structures and partially localized to punctate peroxisome structures (Fig. 3Aa–d); however, knockdown of *MFF* reduced the translocation of DLP1 to the numerous elongated peroxisomes (Fig. 3Ae–h). Furthermore, to investigate whether Mff promotes the translocation of DLP1 to peroxisomes, we transfected *HA_2_-DLP1* into HeLa cells and assessed its intracellular localization 24 h post-transfection. HA_2_-DLP1 was mostly diffused throughout the cytoplasm (Fig. 3Ca–d). By contrast, in cells co-expressing HA_2_-DLP1 and FLAG-Mff, HA_2_-DLP1 colocalized with FLAG-Mff, which is consistent with earlier results (Otera et al., 2010), to Pex14p-positive peroxisomes (Fig. 3Ce–h). Translocation of DLP1 to peroxisomes was not observed in cells co-expressing HA_2_-DLP1 and FLAG-Mff mutants such as MffΔTMD, which lacks a transmembrane domain (TMD), and MffΔN, which lacks amino acids 1–87 including two repeat regions (Fig. 3Ci–p). Next, we assessed the interaction of endogenous Mff and DLP1 by co-immunoprecipitation with Mff-specific antibody. DLP1 was co-immunoprecipitated with Mff from the lysates of HEK293 cells treated with the cross-linker dithiobis[succinimidyl propionate] (DSP) (Fig. 3D), strongly suggesting that endogenous Mff and DLP1 interact. Collectively, these results suggest that Mff recruits DLP1 to peroxisomes.

### Peroxisome elongation is required for Mff-mediated membrane fission

**Fig. 4.**
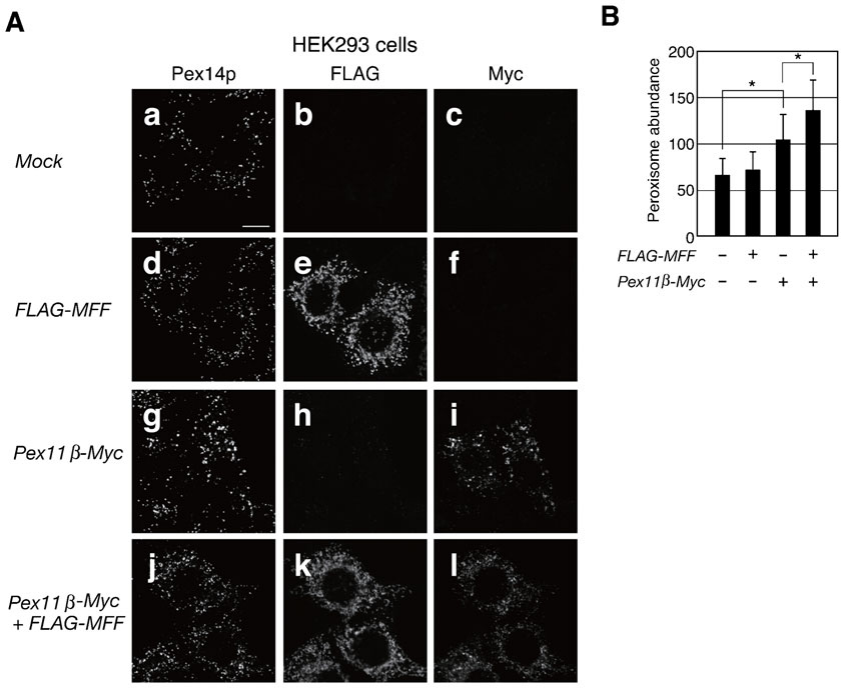
Peroxisomal elongation is required for Mff-mediated membrane fission. (**A**) HEK293 cells were transfected with *FLAG*-*MFF* and *PEX11β-Myc*. After 24 h, cells were stained with antibodies to Pex14p (**a**,**d**,**g**,**j**), FLAG (**b**,**e**,**h**,**k**), and Myc (**c**,**f**,**i**,**l**). Scale bar: 10 µm. (**B**) Peroxisome abundance per cell was measured. Data represent the means ± S.D. of three independent experiments. *P<0.01.

The expression of Mff induces the fragmentation of mitochondria (Otera et al., 2010). To investigate whether the expression of *MFF* induces the proliferation of peroxisomes, we transfected *FLAG-MFF* into HEK293 cells and measured peroxisome abundance. Twenty-four hours post-transfection, imaging results showed that part of the FLAG-Mff-positive particles could be overlaid onto Pex14p-positive peroxisomes, while peroxisome abundance was not significantly altered (Fig. 4Aa–f,B). In addition, we sought to determine whether the elongation of peroxisomes was required for the fission mediated by Mff. *FLAG-MFF* was co-expressed with *PEX11β-Myc* in HEK293 cells. Pex11pβ-Myc induced peroxisomal elongation and a modest increase in peroxisome abundance (Fig. 4Ag–i,B). Furthermore, the number of peroxisomes was more abundant in cells dually expressing Pex11pβ-Myc and FLAG-Mff, resulting in numerous punctate peroxisomes (Fig. 4Aj–l,B</figref>). These results suggest that peroxisomal elongation is required for Mff-mediated peroxisome membrane fission.

### Mff localizes to membrane-constricted regions in elongated peroxisomes

**Fig. 5.**
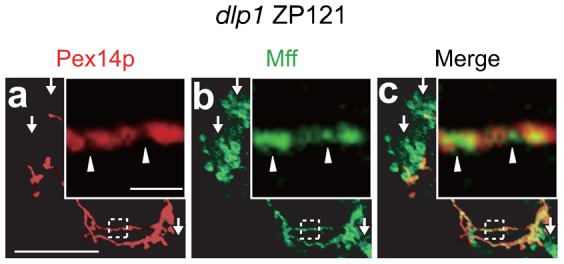
Intracellular localization of endogenous Mff in *dlp1* ZP121 cells. *dlp1* ZP121 cells were immunostained with antibodies to Pex14p (**a**) and Mff (**b**); the merged view of the two proteins is shown (**c**). Scale bar: 10 µm. Insets, higher magnification images of the boxed regions, scale bar: 2 µm. Arrowheads indicate regions enriched for Mff compared to Pex14p-positive regions of peroxisomes; arrows show mitochondria.

Next, we investigated the peroxisomal localization of endogenous Mff in *dlp1* mutant ZP121 CHO cells (Tanaka et al., 2006). ZP121 cells show abnormal tubular peroxisomes due to the expression of a dominant-negative DLP1 mutant; this phenotype permits the assessment of the localization of membrane proteins on elongated peroxisomes. In ZP121 cells, Mff was indeed localized to extended peroxisomes and to mitochondria and partially accumulated in the limited area, which is devoid of Pex14p (Fig. 5). Thus, Mff is localized at the membrane-constriction sites of elongated peroxisomes and functions in peroxisomal fission.

### Pex11pβ, Mff, and DLP1 coordinate peroxisomal fission

**Fig. 6.**
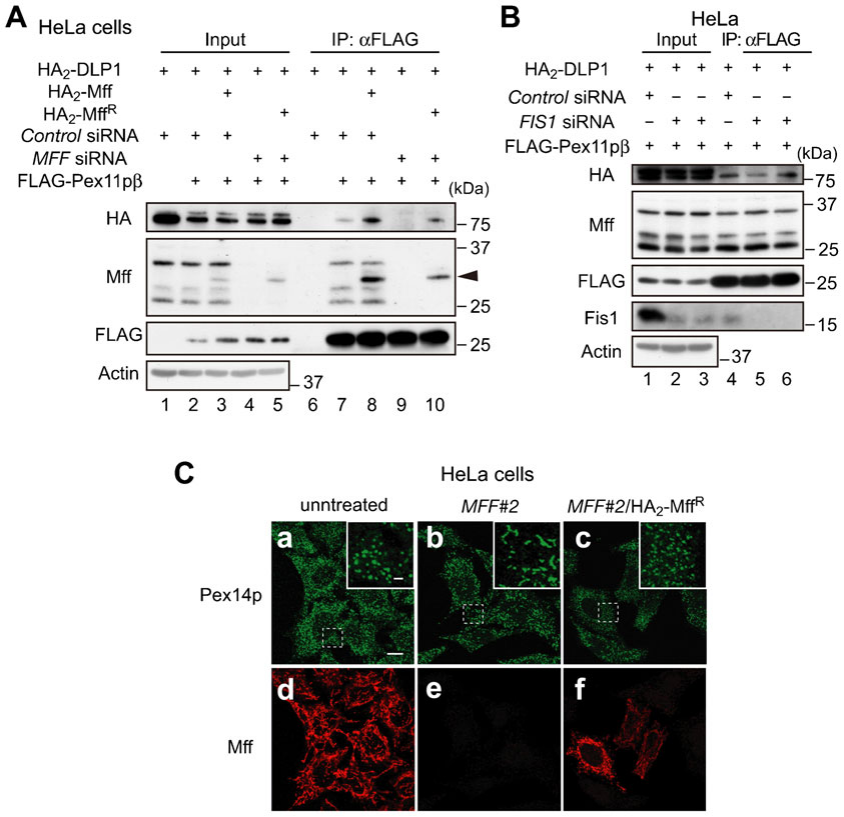
Pex11pβ interacts with DLP1 via Mff. (**A**) After 48 h treatment with control dsRNA or MFF #2 dsRNA, HeLa cells were transfected for 24 h with *FLAG-PEX11β*, *HA_2_*-*DLP1*, *HA_2_*-*MFF*, or *HA_2_*-*MFF^R^*. Cells were then treated with 1 mM DSP, lysed and subjected to immunoprecipitation using anti-FLAG IgG-conjugated agarose. Immunoprecipitates were analyzed by SDS-PAGE and immunoblotting with antibodies to HA, Mff, and FLAG. Arrowhead designates HA_2_-Mff or HA_2_-Mff^R^. Input (10%) was loaded in lanes 1–5. (**B**) HeLa cells treated for 48 h with control dsRNA or two different dsRNAs (*FIS1* #1 and *FIS1* #2) were transfected for 24 h with *FLAG-PEX11β* and *HA_2_*-*DLP1*. Cells were treated with 1 mM DSP and analyzed as in panel A, except that antibodies to HA, Mff, FLAG, and Fis1 were used. Lanes 1–5, input (10%). (**C**) HeLa cells treated for 48 h with *MFF #2* dsRNAs were transfected for 24 h with *HA_2_-MFF^R^*, and stained with antibodies to Pex14p (**a–c**) and Mff (**d–f**). Scale bar: 10 µm. Insets, higher magnification images of the boxed regions, scale bar: 2 µm. Note that peroxisome morphology was restored by the expression of *HA_2_-MFF^R^*.

To address how Pex11pβ, Mff, and DLP1 function during peroxisomal division, we assessed the interaction of Pex11pβ with Mff and DLP1 by immunoprecipitation. Forty-eight hours after adding control or *MFF* dsRNA to HeLa cells, we expressed *FLAG-PEX11β*, *HA_2_*-*DLP1*, *HA_2_*-*MFF,* and siRNA-resistant *HA_2_*-*MFF* (*HA_2_*-*MFF^R^*); immunoprecipitation was then performed with anti-FLAG IgG-conjugated agarose upon DSP treatment. As shown in Fig. 6A, FLAG-Pex11pβ was found to interact with endogenous Mff, HA_2_-Mff, and HA_2_-DLP1, and expression of HA_2_-Mff increased the interplay between FLAG-Pex11pβ and HA_2_-DLP1 (Fig. 6A, lanes 6–8). By contrast, the interplay between FLAG-Pex11pβ and HA_2_-DLP1 was decreased in cells treated with *MFF* dsRNA and restored by the expression of HA_2_-Mff^R^ (Fig. 6A, lanes 9 and 10), indicating that Pex11pβ interacts with DLP1 via Mff. Fis1 was suggested to function in the fission step of peroxisomal division and to form ternary complexes with Pex11pβ and DLP1 (Kobayashi et al., 2007). Thus, we assessed the effect of siRNA targeting *FIS1* on the formation of the complex containing Pex11pβ, Mff, and DLP1. The interplay between FLAG-Pex11pβ and HA_2_-DLP1 was not affected by *FIS1* knockdown, suggesting that Fis1 is not essential for the formation of the Pex11pβ/Mff/DLP1 complex (Fig. 6B). Taken together, these results suggest that Pex11pβ, Mff, and DLP1 cooperate to achieve peroxisome membrane fission.

**Fig. 7.**
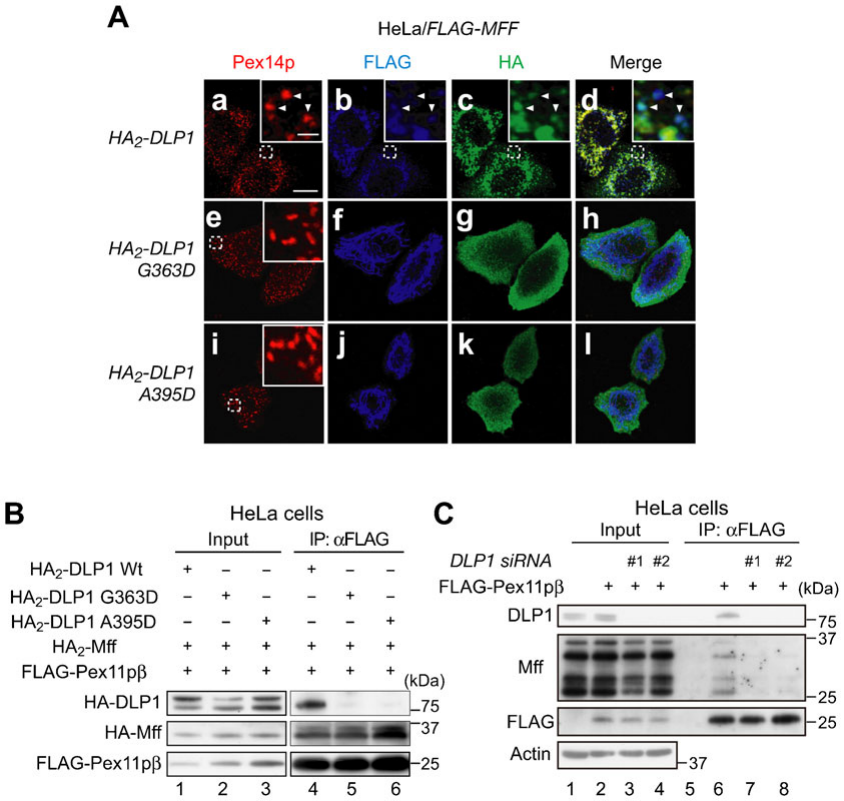
*DLP1* is required for the interaction between Pex11pβ and Mff and the middle domain of DLP1 promotes the formation of the Pex11pβ/Mff/DLP1 complex. (**A**) *FLAG-MFF* was co-expressed with *HA_2_*-*DLP1* (upper panels), *HA_2_*-*DLP1G363D* (middle panels), or *HA_2_*-*DLP1A395D* (lower panels) in HeLa cells. After 24 h, cells were stained with antibodies to Pex14p (**a**,**e**,**i**), FLAG (**b**,**f**,**j**), and HA (**c**,**g**,**k**); the merged view of the three proteins is shown (**d**,**h**,**l**). Scale bar: 10 µm. Insets, higher magnification images of the boxed regions, scale bar: 2 µm. Arrowheads indicate the sites of colocalization of Pex14p, FLAG-Mff, and HA_2_-DLP1. (**B**) HeLa cells were transfected with *HA_2_*-*DLP1*, *HA_2_*-*DLP1-A395D*, or *HA_2_*-*DLP1-G363D*, together with *FLAG-PEX11β* and *HA_2_*-*MFF*. After 24 h, cells were treated with 1 mM DSP. The cell lysates were subjected to immunoprecipitation using anti-FLAG IgG-conjugated agarose and then analyzed by immunoblotting using antibodies to HA and FLAG. Lanes 1–3, input (8%). (**C**) HeLa cells were treated for 48 h with control dsRNA or two different dsRNAs (*DLP1* #1 and *DLP1* #2) and then transfected with *FLAG-PEX11β*. After 24 h, cells were treated with 1 mM DSP. The cell lysates were subjected to immunoprecipitation using anti-FLAG IgG-conjugated agarose and then analyzed by immunoblotting using antibodies to DLP1, Mff, and FLAG, respectively. Input (10%) was loaded in lanes 1–4.

The middle domain of DLP1 is involved in the mitochondrial recruitment and high-order assembly of DLP1 (Chang et al., 2010). Therefore, we assessed whether Pex11pβ forms a complex with the DLP1 middle domain mutants G363D and A395D, both defective in higher-order assembly and GTPase activity (Tanaka et al., 2006; Waterham et al., 2007). As shown in Fig. 7A, the middle domain mutations decreased the translocation of DLP1 to peroxisomes stimulated by the expression of *MFF* in HeLa cells (Fig. 7Ae–l). We transfected *FLAG-PEX11β*, *HA_2_-MFF*, *HA_2_-DLP1*, *HA_2_*-*DLP1 G363D,* and *HA_2_*-*DLP1 A395D* in HeLa cells, and performed immunoprecipitation with anti-FLAG IgG-conjugated agarose after DSP treatment. Wild-type HA_2_-DLP1, but not the HA_2_-DLP1 mutants, was detected in FLAG-Pex11pβ immunoprecipitates (Fig. 7B, lanes 4–6), suggesting that the middle domain of DLP1 is required for the formation of the Pex11pβ/Mff/DLP1 complex. To elucidate the interplay between Pex11pβ, Mff, and DLP1 further, the effect of *DLP1* knockdown on that interaction was assessed. The interplay between FLAG-Pex11pβ and Mff was decreased strikingly in cells treated with *DLP1* dsRNA (#1 and #2) compared to cells treated with control RNAi (Fig. 7C), indicating that DLP1 promotes the interaction between Pex11pβ and Mff. Taken together, it is likely that the complex formed by Mff and DLP1 interacts with Pex11pβ, leading to the formation of large multimeric DLP1 spirals and peroxisome membrane fission.

### Pex11p is not required for the localization of Mff to the membrane-constriction site

**Fig. 8.**
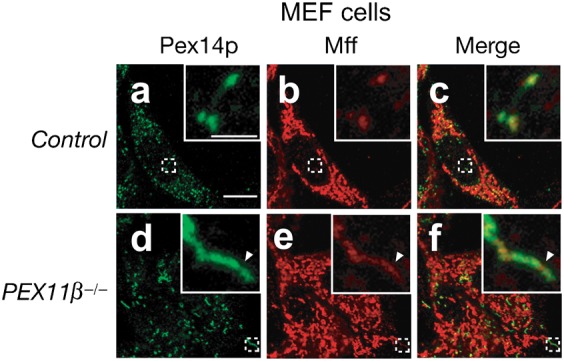
Localization of Mff to the elongated peroxisomes in *PEX11β^−/−^* MEF cells. Control MEF and *PEX11β^−/−^* MEF cells were stained with antibodies to Pex14p (**a**,**d**) and Mff (**b**,**e**); the merged view of the two proteins is shown (**c**,**f**). Scale bar: 10 µm. Insets, higher magnification images of the boxed regions, scale bar: 2 µm. The arrowhead indicates regions enriched for Mff compared to Pex14p-positive regions.

Furthermore, we verified whether Pex11pβ recruits Mff at the membrane-constriction sites, by making use of mouse embryonic fibroblasts (MEF) cells from a *PEX11β*-knocked out mice (Li et al., 2002b) (Fig. 8). In control MEF cells, immunofluorescence straining with Mff antibody showed typical mitochondrial tubular view and dot-like structures with a merged view of Pex14p, suggesting that Mff was localized to both mitochondria and peroxisomes (Fig. 8a–c). In *PEX11β^−/−^* MEF cells, peroxisomes are elongated as previously reported (Li et al., 2002b). Mff was localized to the elongated peroxisomes and with apparent accumulation at the membrane-constriction regions devoid of Pex14p (Fig. 8d–f, arrow), hence implying that Pex11pβ is not essential for the localization of Mff at the membrane-constriction site of elongated peroxisomes.

## Discussion

Mff was identified in an siRNA screen in *Drosophila* cells and shown to be involved in the morphogenesis of mitochondria and peroxisomes (Gandre-Babbe and van der Bliek, 2008). Mff recruits DLP1 to mitochondria (Otera et al., 2010); however, the function of Mff, especially that of endogenous Mff, in peroxisomes remains elusive. In this study, we showed that endogenous Mff localizes to peroxisomes in addition to mitochondria and is responsible for peroxisomal division (Figs 1, 2). Peroxisomal targeting of DLP1 is decreased upon knockdown of *MFF* and is conversely increased by ectopic expression of *MFF* (Fig. 3). Thus, we conclude that Mff recruits DLP1 to peroxisomes.

Pex11p plays a key role in peroxisomal division and mainly supports membrane elongation in peroxisomes (Opaliński et al., 2011; Schrader et al., 1998). In the *PEX11* family, only *PEX11β* is expressed in almost all the types of human cells (Schrader et al., 1998), in contrast to *PEX11α* and *PEX11γ*, which are expressed in a tissue-specific manner (Li et al., 2002a; Schrader et al., 1998). Therefore, understanding Pex11pβ function is key to understanding the mechanisms underlying peroxisome division. In this report, we show that Pex11pβ interacts with DLP1 via Mff (Fig. 6A), suggesting that Pex11pβ forms a ternary complex with Mff and DLP1 during the fission process of peroxisomal division. We reported very recently that Pex11pβ localizes to the constricted regions of elongated peroxisomes, which are devoid of Pex14p (Itoyama et al., 2012). In the present study, we also found that Mff is similarly localized to the constricted regions of elongated peroxisomes (Fig. 5). Therefore, it is likely that the ternary complex comprising Pex11pβ, Mff, and DLP1 promotes fission at the constricted region of elongated peroxisomes. Intriguingly, a recent report showed that Mff stimulates the GTPase activity of DLP1 *in vitro* (Otera and Mihara, 2011), suggesting that the self-assembly of DLP1 is facilitated by Mff. We found here that the middle domain DLP1 mutants, which are defective in self-assembly, decrease DLP1/Mff/Pex11pβ complex formation (Fig. 7B). Taken together, we conclude that DLP1 forms a ternary complex with Mff and Pex11pβ at the constricted regions of elongated peroxisomes; this event is followed by higher-order assembly, resulting in the fission of the peroxisomal membrane.

The interaction of Pex11pβ with Mff was very recently shown by co-immunoprecipitation from cells expressing Flag-tagged Pex11pβ and EGFP-fused Mff (Koch and Brocard, 2012). However, physiological significance of the interaction between Pex11pβ and Mff still remains elusive. A previous study reported that the ectopic expression of *PEX11β* targets DLP1 to peroxisomes (Li and Gould, 2003), implying that Pex11pβ could recruit DLP1 to peroxisomes by interacting with Mff; however, Otera et al. reported that a Mff mutant in which the TMD was replaced with the plasma membrane-targeted CAAX motif elicits the translocation of DLP1 to the plasma membrane (Otera et al., 2010), suggesting that Mff is sufficient to recruit DLP1 to target membranes. Accordingly, it is likely that the interaction between Mff and Pex11pβ is not essential for the recruitment of DLP1 to peroxisomes. Furthermore, we found that knockdown of *DLP1* decreased the interplay between Mff and Pex11pβ. Hence, it is most likely that Mff first interacts with DLP1 and then with Pex11pβ. The mechanisms that regulate the assembly of the peroxisomal fission machinery remain elusive. Pex11pβ interacts with Pex11pγ and Fis1 (Kobayashi et al., 2007; Koch et al., 2010). Based on the observation that overexpression of Pex11pγ induce the membrane elongation and formation of juxtaposed elongated peroxisomes (JEPs) (Koch and Brocard, 2012; Koch et al., 2010), Pex11pγ may protrude the peroxisome membrane. However, the precise role of Pex11pγ remains to be defined. Fis1 is involved in the peroxisomal targeting of DLP1 in yeast and mammalian cells (Kobayashi et al., 2007; Koch et al., 2005; Kuravi et al., 2006). In yeast, Fis1 interacts with Dnm1, the DLP1 homolog, via two adaptor proteins, Mdv1 and Caf4 (Griffin et al., 2005; Tieu and Nunnari, 2000), suggesting that these four proteins are essential for peroxisomal and mitochondrial fission. By contrast, despite the fact that mammalian homologs of Mdv1 and Caf4 have not been identified, direct binding of Fis1 to DLP1 was shown in mammals (Kobayashi et al., 2007). Ectopic expression of *FIS1* indeed induces the proliferation of peroxisomes in a DLP1-dependent manner in COS-7 cells (Koch et al., 2005), probably through increased peroxisomal targeting of DLP1 (Kobayashi et al., 2007). Furthermore, Fis1, Pex11pβ, and DLP1 function together in the fission step of peroxisomal division (Kobayashi et al., 2007). Therefore, there is little doubt that Fis1 promotes the division of peroxisomes in mammalian cells; however, a recent report suggested that normal peroxisome morphology is observed in *FIS1* knockout cells, while abnormally elongated peroxisomes are detected upon knockdown of *DLP1* or *MFF* (Otera et al., 2010). Moreover, the present study showed that the interplay between DLP1 and Pex11pβ was altered in cells treated with siRNA targeting *MFF*, but not *FIS1* (Fig. 6), suggesting that in mammalian cells Fis1 contributes less to peroxisomal morphogenesis than Mff. Collectively, the data show that in mammalian cells Mff plays a key role in peroxisomal fission. At present, we do not know how the interaction between Pex11pβ and Mff/DLP1 is regulated. Mff indeed locates at the membrane-constriction site of the elongated peroxisomes in *PEX11β^−/−^* MEF cells, implying that other factors besides Pex11pβ may be involved in the localization of Mff to the membrane-constriction site of peroxisomes. Very recently, GDAP1 (ganglioside-induced differentiation associated protein 1) was suggested to be required for peroxisome fission at the downstream of Pex11pβ and the upstream of fission steps mediated by Mff and DLP1 (Huber et al., 2013), inferring that GDAP1 likely mediates the interaction between Pex11pβ and Mff/DLP1 complex.

The regulatory mechanism underlying peroxisomal division remains elusive. In mitochondria, Fis1, Mff, MiD49, and MiD51 can each recruit DLP1 in one of the rate-limiting steps of mitochondrial fission (Cereghetti et al., 2008; Losón et al., 2013; Otera and Mihara, 2011; Palmer et al., 2011; Zhao et al., 2011). For instance, overexpression of *MFF* facilitates the mitochondrial targeting of DLP1, resulting in the fragmentation of mitochondria (Otera et al., 2010). By contrast, peroxisomal proliferation, resulting from peroxisomal fission, was not increased by the overexpression of *MFF* despite massive recruitment of DLP1 to the peroxisomal membrane (Fig. 3C, Fig. 4). Moreover, the proliferation of peroxisomes is significantly suppressed in *AOx*-deficient fibroblasts, although DLP1 localizes to peroxisomes (Itoyama et al., 2012). These findings strongly suggest that the recruitment of DLP1 is not a rate-limiting step for peroxisomal division. Interestingly, the fission of peroxisomes in the methylotrophic yeast *Pichia pastoris* is regulated by the interaction of Pex11p with Fis1 via phosphorylation of Pex11p in oleate medium, not methanol (Joshi et al., 2012). In mammalian cells, DHA is one of the mediators of peroxisomal division and induces elongation of peroxisomes in a Pex11pβ-dependent manner (Itoyama et al., 2012). Here we demonstrated that DHA promotes the proliferation of peroxisomes in *AOx*-deficient fibroblasts in a manner dependent on Mff and DLP1 (Fig. 2). Furthermore, co-expression of *MFF* and *PEX11β*, but not expression of *MFF* alone, promotes peroxisomal proliferation (Fig. 4). These data suggest that the elongation of peroxisomes, giving rise to the formation of Pex11pβ-enriched and membrane-constricted regions (Itoyama et al., 2012), is a prerequisite for peroxisomal fission via activation of DLP1. Taken together, the membrane elongation of peroxisomes is likely to be a rate-limiting step in peroxisomal division and might facilitate the formation of the DLP1 spiral structures at the constricted regions, leading to division.

## Materials and Methods

### Cell culture and DHA supplementation

Human skin fibroblasts from a healthy subject (Tig120) were purchased from the Human Science Research Resources Bank (Osaka, Japan). Fibroblasts from a patient with *AOx* deficiency (PDL30092) were described previously (Ferdinandusse et al., 2007; Poll-The et al., 1988). Control MEF and *PEX11β^−/−^* MEF cells were a generous gift from Dr S. J. Gould (Li et al., 2002b). Fibroblasts, MEF, HeLa cells, and HEK293 cells were cultured at 37°C in Dulbecco's modified Eagle medium (DMEM; GIBCO BRL, Rockville, MD) supplemented with 10% fetal calf serum (FCS; SIGMA, St. Louis, MO) in 5% CO_2_ (Okumoto et al., 1998). CHO cell lines, including CHO-K1 and *dlp1* ZP121 (Tanaka et al., 2006) cells, were cultured as described previously (Tsukamoto et al., 1990). DHA (Nacalai Tesque, Kyoto, Japan) dissolved in DMEM supplemented with 0.4% fatty acid-free bovine serum albumin (Nacalai Tesque) was used in cell cultures at a final concentration of 150 µM as described previously (Itoyama et al., 2012).

### Antibodies

The antibodies used were rabbit antiserum to rat Pex14p (Shimizu et al., 1999), HA peptide (Otera et al., 2000), and guinea pig antiserum to rat Pex14p (Mukai et al., 2002). Polyclonal antibodies to FLAG and human Fis1 were purchased from Sigma and Enzo Life Sciences (Farmingdale, USA), respectively. Monoclonal antibodies to human DLP1 (BD Biosciences, Franklin lake, NJ), human Tom20 (Santa Cruz Biotech, Santa Cruz, CA), P450 reductase (Santa Cruz Biotech), actin (Chemicon, Temecula, CA), HA (Covance, Princeton, NJ), and c-Myc (Santa Cruz) were purchased.

Rabbit antiserum against human Mff was raised as follows: an expression vector encoding the primary sequence (residues at 27–173) of human (*Hs*) Mff fused to GST, termed GST-HsMff (27–173), was constructed as previously described (Shimizu et al., 1999) using *FLAG-MFF* as a template and the primers GST-HsMff (27–173)-Fw 5′-CGCGGATCCATGGCAGAAATTAGTCGAATT-3′ and GST-HsMff (27–173)-Rv 5′-CAAGCGGCCGCCCATACAGAGAATCATTTC-3′. A BamHI-EcoRI fragment of the PCR product was ligated into the corresponding sites of pGEX6P-1 (GE Healthcare, Chalfont, UK). *Escherichia coli* BL21 cells were transformed with pGEX6P-1-*GST-HsMFF (27–173)* and grown according to the manufacturer's instructions. BL21 cells were then harvested in lysis buffer (1% Triton X-100, 1 mM phenymethylsulfonyl fluoride in PBS) and sonicated. The lysate was centrifuged at 20,000 × *g* for 10 min and the supernatant was subjected to purification using glutathione-Sepharose beads (GE Healthcare). After a thorough wash, purified GST-Mff (27–173) was cleaved with PreScission protease (GE Healthcare) to remove the GST moiety, and the eluted Mff (27–173) was further purified with Q-Sepharose Fast Flow ion-exchanger columns (GE Healthcare). The peak fractions were recovered as recombinant HsMff (27–173). The rabbit anti-Mff antibody was raised by conventional subcutaneous injection of HsMff (27–173) in PBS/0.1% Triton X-100 (Okumoto et al., 1998).

### RNA interference

For RNAi, six sets of complementary antisense oligonucleotides were designed (Invitrogen, Carlsbad, CA). The sequences were as follows: human *MFF* #1 oligonucleotides 5′-UUAUCACACUAGCAUUUGGAACUCC-3′ and 5′-GGAGUUCCAAAUGCUAGUGUGAUAA-3′; human *MFF* #2 oligonucleotides 5′-UAUAUUUGAAAUGCCAUACCUGACG-3′ and 5′-CGUCAGGUAUGGCAUUUCAAAUAUA-3′; human *FIS1* #1 oligonucleotides 5′-UUACGGAUGUCAUCAUUGUACUUGC-3′ and 5′-GCAAGUACAAUGAUGACAUCCGUAA-3′; human *FIS1* #2 oligonucleotides 5′-UAAUCCCGCUGUUCCUCCUUGCUCC-3′ and 5′-GGAGCAAGGAGGAACAGCGGGAUUA-3′; human *DLP1* #1 oligonucleotides 5′-AAACCUCAGGCACAAAUAAAGCAGG-3′ and 5′-CCUGCUUUAUUUGUGCCUGAGGUUU-3′; human *DLP1* #2 oligonucleotides 5′-AUUUGAGGCAGCUGGAUGAUGUCGG-3′ and 5′-CCGACAUCAUCCAGCUGCCUCAAAU-3′. Fibroblasts and HeLa cells were transfected twice after a 24 h interval with dsRNA at a concentration of 40 nM using Lipofectamine 2000 (Invitrogen).

### DNA construction

For *FLAG-MFF*, *FLAG-MFFΔTMD*, *HA_2_-MFF*, and *HA_2_-DLP1*, human *MFF* (splice variant 8) (Gandre-Babbe and van der Bliek, 2008) and *DLP1* cDNA were amplified by RT-PCR using total RNA isolated from HeLa cells and the primer pairs described below; MFF-Fw 5′-AGTGGATCCGGATGGCAGAAATTAGTCGAATTCAGTACG-3′, MFF-Rv 5′-CAAGCGGCCGCGCGGCGAAACCAGAGCCAG-3′, MFFΔTM-Rv 5′-GTTGCGGCCGCCATTTCTCTTTTAGCACG-3′, MFFΔN-Rv 5′-AAAGGATCCGGAATAATGAAGATGTTTCATTTTC-3′, DLP1-Fw 5′-AAGGATCCGGATGGAGGCGCTAATTCCTGT-3′, and DLP1-Rv 5′-AAGCGGCCGCTCACCAAAGATGAGTCTCCC-3′. PCR products were cloned into pcDNA3.1 Zeo^+^/FLAG-Ubiquitin (Okumoto et al., 2011b) or pcDNA3.1 Zeo^+^/HA_2_-Ubiquitin (Okumoto et al., 2011a) by replacing the BamHI-NotI fragments of vectors. We also used pUcD2Hyg/*FLAG-PEX11β* (Abe and Fujiki, 1998) and pEF/*PEX11β-Myc* (Y.Y. and Y.F., unpublished).

Site-directed mutagenesis was performed to introduce substitutions in *DLP1* and *MFF* using the following primers: DLP1 G363-Fw 5′-AACTTCGGAGCTATGCGGTGATGCTAGAATTTGTTATATTT-3′, DLP1 G363D-Rv 5′-AAATATAACAAATTCTAGCATCACCGCATAGCTCCGAAGTT-3′, DLP1 A395D-Fw 5′-CACTCTTGACATTTTGACTGACATTAGAAATCATACTGGTC-3′, DLP1A395D-Rv 5′-GACCAGTAGCATTTCTAATGTCAGTCAAAATGTAATAGTG-3′, MFF^R^-Fw 5′-ACAAGGATTCCAAGAAGGAGTTCCAAATGCTAGTGTGATAATGCAAGTTCCGGAGAG-3′, and MFF^R^-Rv 5′-CTCTCCGGAACTTGCATTATCACACTAGCATTTGGAACTCCTTCTTGGAATCCTTGT-3′.

### Morphological analysis

Cells were fixed with 4% paraformaldehyde (pH 7.4) for 15 min at room temperature. Peroxisomes were visualized by indirect immunofluorescence staining with the indicated antibodies as described previously (Mukai et al., 2002). Antigen–antibody complexes were detected with goat anti-mouse and anti-rabbit IgG conjugated to Alexa Fluor 488 or Alexa Fluor 568 (Molecular Probes, Eugene, OR). Cells were observed under a fluorescence light microscope (Axioplan2) and by confocal laser microscopy (LSM710; Carl Zeiss, Oberkochen, Germany).

The number of peroxisomes was counted in at least 50 randomly selected cells (Kim et al., 2006). Optical images obtained by confocal fluorescence microscopy were converted into threshold images and then the number of peroxisomes was calculated using the Particle Analysis package of ImageJ. Values are means ± S.D. of three independent experiments.

### Sedimentation analysis

HeLa, HEK293, and CHO-K1 cells were harvested in homogenization buffer (10 mM Hepes-KOH, pH 7.4, 250 mM sucrose, 1 mM EDTA, and protease inhibitor cocktail), homogenized with a Potter-Elvehjem homogenizer and centrifuged at 800 × *g* for 5 min to remove nuclei. The post-nuclear supernatant (PNS) fraction was centrifuged at 100,000 × *g* for 30 min to obtain cytosol and organelle fractions.

For subcellular fractionation, control fibroblasts were homogenized in homogenization buffer (10 mM Hepes-KOH, pH 7.4, 250 mM sucrose, and protease inhibitors cocktail), and centrifuged at 1,000 × *g* for 10 min to yield the PNS fraction. The PNS fraction was subsequently centrifuged at 2,500 × *g* for 10 min to obtain a post-heavy mitochondrial (PHM) fraction. The PHM fraction was incubated with 1 mM puromycin and 500 mM KCl for 30 min on ice to strip ribosomes from the rough ER (Walter and Blobel, 1983), and subjected to ultracentrifugation in 19.5% Opti-prep density gradient in a Beckman NVT65.2 rotor (Beckman Instruments, Fullerton, CA) at 46,000 rpm for 3 h (Honsho et al., 2008). The gradient was fractionated into 12 tubes.

### Immunoprecipitation

For immunoprecipitation using anti-Mff antibody, HEK293 cells were treated for 30 min at room temperature with 0.5 mM DSP. The cross-linking reaction was then quenched by incubation in 0.1 mM Tris-HCl (pH 7.4) for 15 min at room temperature as described previously (Kobayashi et al., 2007). Cells were lysed in immunoprecipitation buffer (20 mM Hepes-KOH, pH 7.4, 150 mM NaCl, 0.5% CHAPS, protease inhibitor cocktail). The lysate was incubated at 8°C for 15 min and then centrifuged at 20,000 × *g* for 10 min. The supernatants were subjected to immunoprecipitation with anti-Mff antibody as described previously (Kobayashi et al., 2007).

For immunoprecipitation using anti-FLAG IgG-conjugated agarose (Sigma), HeLa cells were treated for 30 min at room temperature with 1 mM DSP. After quenching with 50 mM Tris-HCl (pH 7.4) for 30 min at room temperature, cells were lysed in immunoprecipitation buffer (20 mM Hepes-KOH, pH 7.4, 150 mM NaCl, 1% CHAPS, and protease inhibitor cocktail). The lysate was incubated at 4°C for 30 min and then centrifuged at 20,000 × *g* for 10 min. The supernatants were subjected to immunoprecipitation with anti-FLAG IgG-conjugated agarose.
